# Assessment of Five Chilling Tolerance Traits and GWAS Mapping in Rice Using the USDA Mini-Core Collection

**DOI:** 10.3389/fpls.2017.00957

**Published:** 2017-06-08

**Authors:** Michael R. Schläppi, Aaron K. Jackson, Georgia C. Eizenga, Aiju Wang, Chengcai Chu, Yao Shi, Naoki Shimoyama, Debbie L. Boykin

**Affiliations:** ^1^Department of Biological Sciences, Marquette University, MilwaukeeWI, United States; ^2^Dale Bumpers National Rice Research Center, United States Department of Agriculture – Agricultural Research Service, StuttgartAR, United States; ^3^State Key Laboratory of Plant Genomics, Institute of Genetics and Developmental Biology, Chinese Academy of SciencesBeijing, China; ^4^United States Department of Agriculture – Agricultural Research Service, StonevilleMS, United States

**Keywords:** rice chilling tolerance, chilling acclimation, abiotic stress, GWAS based QTL mapping, rice mini-core, japonica rice, indica rice

## Abstract

Rice (*Oryza sativa* L.) is often exposed to cool temperatures during spring planting in temperate climates. A better understanding of genetic pathways regulating chilling tolerance will enable breeders to develop varieties with improved tolerance during germination and young seedling stages. To dissect chilling tolerance, five assays were developed; one assay for the germination stage, one assay for the germination and seedling stage, and three for the seedling stage. Based on these assays, five chilling tolerance indices were calculated and assessed using 202 *O. sativa* accessions from the Rice Mini-Core (RMC) collection. Significant differences between RMC accessions made the five indices suitable for genome-wide association study (GWAS) based quantitative trait loci (QTL) mapping. For young seedling stage indices, *japonica* and *indica* subspecies clustered into chilling tolerant and chilling sensitive accessions, respectively, while both subspecies had similar low temperature germinability distributions. *Indica* subspecies were shown to have chilling acclimation potential. GWAS mapping uncovered 48 QTL at 39 chromosome regions distributed across all 12 rice chromosomes. Interestingly, there was no overlap between the germination and seedling stage QTL. Also, 18 QTL and 32 QTL were in regions discovered in previously reported bi-parental and GWAS based QTL mapping studies, respectively. Two novel low temperature seedling survivability (LTSS)–QTL, *qLTSS3-4* and *qLTSS4-1*, were not in a previously reported QTL region. QTL with strong effect alleles identified in this study will be useful for marker assisted breeding efforts to improve chilling tolerance in rice cultivars and enhance gene discovery for chilling tolerance.

## Introduction

In high altitude environments or temperate climates, like that found in the rice growing regions of the United States, Asian rice (*Oryza sativa* L.) is often exposed to low or chilling temperatures, below 10°C, during the spring planting season when rice seed is directly planted into the soil (drill-seeded) or pre-germinated seed is sown in fields flooded with cool irrigation water (water-seeded) as compared to transplanting older seedlings. For instance, average low temperatures for the months when rice is planted in the Sacramento Valley of California (April to May) or in Arkansas (March to April) are 9.3 and 8.8°C, respectively, but daily minima can be even lower^1^. Having improved chilling tolerance at the germination stage would allow drill-seeded rice to be planted earlier in the season, thus allowing the rice crop to take advantage of the usually abundant rainfall that occurs early in the growing season. Once germinated, rice seedlings are often exposed to periods of alternating warm and chilling temperatures, which can affect the survivability of the seedlings depending on the level of chilling tolerance. In rice, as in other crop plants, chilling stress is known to be a complex, quantitative trait controlled by multiple genes which is often divided into tolerance at the germination, seedling and reproductive stages (da Cruz et al., 2013; Zhang et al., 2014a).

Cold temperatures can lead to severe osmotic and oxidative stress in plants (Wang et al., 2003; Yun et al., 2010). Although rice is chilling sensitive due to its subtropical origin, very few genes that could be used to improve chilling tolerance have been identified over the last 20 years (da Cruz et al., 2013; Zhang et al., 2014a). Of those, *CTS12* was the first gene identified to be associated with a “*Cold Tolerance of the Young Seedling*” (*CTS*) quantitative trait locus (QTL) and codes for a stress-induced protein with multifunctional glutathione transferase (GST) activity (Takesawa et al., 2002; Kim et al., 2011). A single I > V amino acid substitution in GST correlates with reduced *in vitro* activity of the enzyme from chilling sensitive plants. Similarly, *qLTG3-1*, the first gene identified to associate with a “*Low-Temperature Germinability*” (*LTG*) QTL, codes for a secreted hybrid glycine-rich protein, and a single nucleotide substitution differentiates between strong and weak alleles (Fujino et al., 2008). The biochemical function of the *qLTG3-1* product is not known; however, a conserved gene in *Arabidopsis* was previously shown to improve freezing tolerance and germinability under different stress conditions (Bubier and Schläppi, 2004; Zhang and Schläppi, 2007; Xu et al., 2011). *Ctb-1*, the first gene to associate with a “*Cold Tolerance at the Booting Stage*” (*CTB*) QTL, codes for a F-box protein, suggesting that a ubiquitin-proteasome pathway is involved in chilling tolerance at this stage (Saito et al., 2010). Moreover, the first gene presumably involved in signal transduction, “*Chilling-Tolerance Divergence 1*” (*COLD1*), was recently identified and codes for a G-protein signaling regulator leading to Ca^++^ influx into cells of chilling tolerant cultivars (Ma et al., 2015). To better understand chilling tolerance, it is essential to identify additional rice chilling tolerance genes.

There is considerable genetic variation in cultivated self-pollinating plants such as rice, which was previously explored for chilling tolerance QTL mapping and gene discovery (e.g., Hou et al., 2004; Ji et al., 2009; Zeng et al., 2009; Shinada et al., 2013; reviewed in da Cruz et al., 2013; Zhang et al., 2014a). Altogether, there are over 60 previously reported genomic regions on all 12 rice chromosomes containing QTL for chilling tolerance at the germination and young seedling stage, a majority of which were obtained repeatedly by various research groups. Most previously published data were obtained using bi-parental mapping populations from *O. sativa* ssp. *Indica* ×*O. sativa* ssp. *japonica* crosses, because the *japonica* subspecies is more chilling tolerant than *indica* (Mackill and Lei, 1997; da Cruz and Milach, 2004; Mao and Chen, 2012; Ma et al., 2015). While bi-parental mapping populations are an important tool for gene discovery, the amount of genetic background is limited to the parental lines used. These studies do not account for the genetic diversity in rice beyond the parental lines and are limited almost exclusively to the use of a few mapping populations generated from East Asian cultivars representing the *indica* and *japonica* subspecies.

Recently, chilling tolerance at the germination (Fujino et al., 2015; Shakiba et al., 2017), seedling (Pan et al., 2015; Lv et al., 2016; Wang et al., 2016a) and reproductive (Pan et al., 2015; Zhu et al., 2015; Shakiba et al., 2017) stages was mapped using genome-wide association study (GWAS) analyses with several different collections of rice accessions genotyped with either SSR (single sequence repeat) markers or SNP (single nucleotide polymorphism) markers. At the germination stage, GWAS mapping of a collection of 63 rice varieties from Hokkaido, Japan identified 17 QTL (Fujino et al., 2015) and evaluation of the Rice Diversity Panel 1 (RDP1) revealed a total of 42 QTL (Shakiba et al., 2017). RDP1 seedlings at the three-leaf stage, were subjected to 3 days of chilling treatment, scored for chilling survivability and GWAS mapping revealed 67 QTL located on 11 chromosomes (Wang et al., 2016a). A mini-core of 174 Chinese rice accessions with 5 mm coleoptiles was cold treated for 10 days, scored for chilling recovery and GWAS mapping detected 22 QTL for chilling tolerance (Pan et al., 2015). Lastly, evaluation of a large collection of 529 rice accessions, including 202 accessions from the China Core Collection and 327 from the World Core Collection, grown under natural winter time chilling conditions in Wuhan, China and cold shock stress conditions at the four-leaf seedling stage identified 132 loci associated with at least one of these measures or the associated electrolyte leakage measurements (Lv et al., 2016). Haplotype analysis of these accessions for the *OsMYB2* gene involved with chilling tolerance, revealed the *indica-japonica* subspecies differentiation, with the *japonica* subspecies being more chilling tolerant and having a wider latitudinal distribution (Lv et al., 2016).

The accessions in the rice mini-core collection (RMC) were selected to represent the diversity in the USDA rice collection composed of 18,709 accessions obtained from 114 countries (Agrama et al., 2009). The RMC consists of 217 rice accessions originating from Africa (17 accessions), Australia (1), Central Asia (14), Central America (10), China (20), Eastern Europe (9), the Mideast (5), North America (6), the North Pacific (11), South America (14), the South Pacific (24), Southeast Asia (21), South Asia (41), Western Europe (9) and the origin of one accession is unknown (Li et al., 2010). Genotypic analysis revealed the five major rice subpopulation groups are represented in this collection with the *japonica* subspecies (hereafter referred to as *JAPONICA*) composed of the *aromatic* subpopulation (6 accessions), *tropical japonica* (33), and *temperate japonica* (34) and the *indica* subspecies (hereafter referred to as *INDICA*) composed of *aus* (38) and *indica* (68). Of the remaining 38 accessions, 24 were *O. sativa* accessions classified as an admixture of two or more subpopulation groups and 14 accessions represented other *Oryza* species, thus were not *O. sativa* (Li et al., 2010; personnel communication). This rich genetic diversity makes the RMC attractive for GWAS mapping, as demonstrated by the marker-trait associations already reported for grain yield and other harvest index components (Li et al., 2011), silica concentration (Bryant et al., 2011), harvest index (Li et al., 2012), resistance to rice sheath blight disease (Jia et al., 2012), protein content (Bryant et al., 2013), and pericarp color (Wang et al., 2016b).

To further investigate the genetic diversity for chilling tolerance at the germination and young seedling stages, we evaluated 202 *O. sativa* accessions from the RMC using two assays for the germination stage and three assays for the seedling stage. From these assays, the following five chilling tolerance indices were calculated; low temperature germinability (LTG), plumule growth rate after cold germination (PGCG), low temperature seedling survivability (LTSS), plumule recovery growth after cold exposure (PGC) and low temperature survival (LTS). Subsequent GWAS mapping for these five indices allowed us to identify novel chilling tolerance QTL and accessions with superior seedling chilling tolerance, which can be used to incorporate improved chilling tolerance into elite rice breeding lines.

## Materials and Methods

### Rice Mini-Core (RMC) Materials

Seed (including the lemma and palea) of 202 RMC accessions classified as *O. sativa* was obtained from the Genetic Stocks-*Oryza* (GSOR) collection located at the USDA-ARS Dale Bumpers National Rice Research Center (USDA-ARS DBNRRC; Stuttgart, AR). The 202 RMC accessions used in this study represented the *aromatic* subpopulation (6 accessions), *tropical japonica* (33), *temperate japonica* (34), *aus* (38), *indica* (68), and 23 accessions were an admixture of two or more subpopulation groups (Li et al., 2010; personnel communication).

### Indices for Evaluating Chilling Tolerance

To screen RMC accessions for different chilling tolerance abilities, growth chamber/incubator based assays were done to mimic the early spring (March to April) field-growing environments of Arkansas and California, two major rice producing areas of the United States, using the following five indices:

#### Low Temperature Germinability (LTG)

**FIGURE 1:**
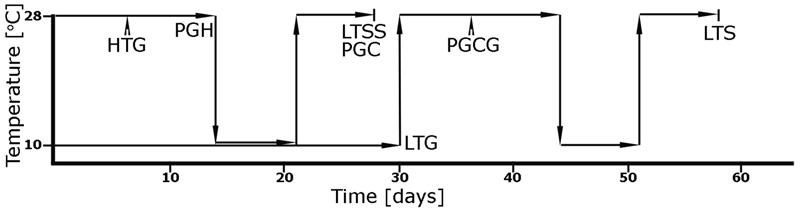
Schematic diagram of temperature changes and growth periods used for the five chilling tolerance indices assessed in this study. *Low Temperature Germinability* (LTG): Mean percent germination of seeds after 30 days of incubation at 10 ± 1°C was normalized with the mean percent germination of seeds at 28 ± 1°C (high temperature germination index, HTG). *Plumule Growth rate after Cold Germination* (PGCG): After 30 days of germination at 10 ± 1°C, seeds were incubated at 28 ± 1°C for 4 days, and (mean plumule length at day 4 minus mean coleoptile length) was divided by 4. *Low Temperature Seedling Survivability* (LTSS): After 2 weeks of growth at 28 ± 1°C, healthy seedlings were incubated for 1 week at 10 ± 1°C, then again at 28 ± 1°C for 1 week of recovery growth, and (the mean number of green and healthy looking seedling after 1 week of recovery growth divided by the number of the initially healthy looking 2-week-old seedlings) was multiplied by 100. *Plumule recovery Growth after Cold exposure* (PGC): The mean leaf length of seedlings after 2 weeks of growth at 28 ± 1°C was recorded as the Plumule growth at High temperature (PGH) index, then again measured after incubation for 1 week at 10 ± 1°C and 1 week of recovery growth at 28 ± 1°C. The mean plumule length after 1 week of recovery growth was normalized by subtraction of PGH and division of that number by PGH and multiplied by 100. *Low Temperature Survival* (LTS): After 30 days of germination at 10 ± 1°C, seeds were incubated for 2 weeks at 28 ± 1°C followed by selection of healthy seedlings, which were incubated for 1 week at 10 ± 1°C, then for 1 week of recovery growth at 28 ± 1°C, and (the mean number of green and healthy looking seedling after 1 week of recovery growth divided by the number of the initially healthy looking 2-week-old seedlings) was multiplied by 100.

For LTG measurements, germination was defined as visible coleoptile emergence through the lemma and palea (hull). Seeds were incubated on water soaked filter paper in Petri dishes, 30–50 per dish, at 10 ± 1°C with a 10-h day/14-h dark photoperiod and 165 μE photon flux in an AR-66L growth chamber (Percival, IA). The Low Temperature-uncorrected (LTG-u) index was defined as percent germination after 30 days at 10°C. The 10°C temperature point was chosen because it is at the threshold of survivability for young rice seedlings (reviewed in da Cruz et al., 2013). For each accession, duplicate plates were randomly distributed in the Percival growth chamber to control for environmental differences. Chilling tolerant check, Italica Livorno (*temperate japonica*; Fujino et al., 2008), and chilling sensitive check, Zhenshan 97 (*indica*), all non-RMC accessions, were used as controls. For each trial, mean LTG-u scores from two Petri dishes were recorded and normalized with the mean percent germinability of seeds at 28°C, defined as the high temperature germination index HTG. The LTG index was calculated as (LTG-u divided by HTG) times 100 (**Figure 1**).

Low-temperature germinability was evaluated in 2010 (two replications), 2011 (one replication) and 2012 (one replication). For statistical analysis, the zero values for LTG, in other words, the accessions which exhibited no chilling tolerance, were replaced with 0.25. The generalized linear mixed model in SAS (SAS Institute Inc., 2012), procedure GLIMMX, was used to compute the least squares means (LSmeans). For LSmeans estimated across years, year was treated as a block effect and the individual accessions were treated as a fixed effect. These adjusted LSmeans were used for the GWAS mapping.

#### Plumule Growth Rate after Cold Germination (PGCG)

To determine PGCG, Petri dishes with 30 rice seeds each, were first germinated at 10 ± 1°C with a 10-h day/14-h dark photoperiod and 165 μE photon flux in an AR-66L growth chamber for 30 days (similar to LTG), coleoptile lengths of 10 randomly selected seeds were recorded. Plumule lengths were then measured 4 days after recovery at 28 ± 1°C and averaged. Mean plumule growth rates after cold germination of 10 young seedlings from two randomly distributed Petri dishes were calculated as PGCG, (mean plumule length at day 4 minus mean coleoptile length at day 0) divided by 4. This value reflected the growth rate of the coleoptile/plumule over an initial 4-day-period (**Figure 1**). Chilling tolerant check, Italica Livorno, and chilling sensitive check, Zhenshan 97 were used as controls. The PGCG trail was conducted one time with duplicate sampling and arithmetic means were calculated for GWAS mapping.

#### Low Temperature Seedling Survivability (LTSS)

For LTSS measurements, rice seedlings were grown at 28 ± 1°C with a 10-h light period and 165 μE photon flux in an AR-66L growth chamber for 2 weeks in Petri plates on water-soaked cheese cloth, 30–50 plants per plate, until they reached approximately the 2-leaf-stage (V2; Counce et al., 2000). After 2 weeks, seeds that failed to germinate or grow were removed and the number of healthy seedlings recorded. The mean number of healthy seedlings used for each LTSS trial was 25.6 ± 3.6 (SD), specifically: 22.4 (admix); 25.9 (*aro*); 26.6 (*aus*); 27.1 (*indica*); 23.2 (*temperate japonica*); and 26.0 (*tropical japonica*). The temperature was then lowered to 10 ± 1°C for 1 week and raised again to 28 ± 1°C for 1 week of recovery growth. The mean percent LTSS for each accession was calculated as LTSS, (number of green and healthy looking seedlings after 1 week of recovery growth divided by the number of initial healthy looking seedlings) times 100 (**Figure 1**). The chilling tolerant check Italica Livorno, and either chilling sensitive checks Zhenshan 97 or IR65 (*indica*), all non-RMC accessions, were used as controls.

Low temperature seedling survivability was measured as five different trials conducted twice in 2011 (2011a and 2011b), once in 2012, 2015, and 2016. The 2011a trial had duplicate sampling, thus the overall mean was weighted to account for the duplicate sampling. For statistical analysis, the zero values were replaced with 0.25, as discussed for LTG, for the single year data and for the combined years, LSmeans were estimated across years as described for LTG.

#### Plumule Recovery Growth after Cold Exposure (PGC)

To determine PGC, the mean leaf length of 2-week old seedlings at the V2 stage, before cold exposure, was recorded, and then again after 1 week of recovery growth at 28 ± 1°C following a 1-week chilling stress treatment at 10 ± 1°C. Both treatments were done with a 10-h day/14-h dark photoperiod and 165 μE photon flux in an AR-66L growth chamber. The mean percent leaf recovery growth rate after cold exposure was calculated as PGC, [(plumule length after 1 week of recovery growth minus plumule length before cold exposure) divided by plumule length before cold exposure] times 100 (**Figure 1**). Chilling tolerant check Italica Livorno and either chilling sensitive check, Zhenshan 97 or IR65, were used as controls. Dead seedlings were excluded from measurements and if all seedlings of one accession died during the recovery period, a value of zero was recorded.

Plumule recovery growth after cold exposure was measured in three different trials conducted in 2010, 2011, and 2016. The procedure GLIMMX was used to estimate LSmeans averaged across years. Year was treated as a block type replication. Data was transformed using the log of PGC + 1 prior to analysis. For statistical analysis, the zero values were replaced with 0.25.

#### Low Temperature Survival (LTS)

The mean percent of overall LTS for each accession was determined after two cold stress treatments, one during germination, and the other at the seedling stage. First, rice seeds were germinated at 10 ± 1°C for 30 days (similar to LTG) followed by 2 weeks of growth to the V2 stage at 28 ± 1°C. Second, healthy looking, approximately 2-leaf stage seedlings, were selected and incubated for 1 week at 10 ± 1°C followed by 1 week of recovery at 28 ± 1°C (similar to LTSS). The mean number of healthy seedlings used for each LTS trial was 27.2 ± 9.2 (SD), specifically: 21.4 (admix); 32.3 (*aro*); 25.0 (*aus*); 25.3 (*indica*); 29.8 (*temperate japonica*); and 34.5 (*tropical japonica*). The mean percent of overall LTS for each accession was calculated as LTS, (number of green seedlings after 1-week recovery of growth divided by the number of initial healthy seedlings) times 100 (**Figure 1**). Chilling tolerant check, Italica Livorno, and either chilling sensitive check, Zhenshan 97 or IR65, were used as controls.

Low temperature seedling survivability was measured in two trials conducted in 2011 and 2012. For statistical analysis, procedure GLIMMX was used to estimate means averaged across years. Year was treated as a block type replication. A binomial distribution with a logit link was used to describe the survival. Prior to analysis, the zero values were replaced with 0.25.

### Correlation Coefficient

The multi-year LSmeans for LTG, LTSS, PGC and LTS, and multi-year arithmetic mean for PGCG, were used to calculate the Pearson’s correlation coefficients *r* between the five indices. The PEARSON function of Microsoft Excel was used to calculate the *r* coefficients. The coefficients *r* were transformed to *t* using the formula *t* = *r*^∗^SQRT(*N*-2)/SQRT(1-*r*^2^) in Excel where *N* was the number of observations. The TDIST function of Excel was used to calculate *p* from *t*.

### Genome-Wide Association Study (GWAS) Analyses

The GWAS mapping was conducted with 157 markers including 148 SSR markers, three InDel markers and six SNP markers (Bryant et al., 2011). Prior to conducting the GWAS based mapping analyses, the population structure was examined using the model-based program STRUCTURE (Pritchard et al., 2000) and Principal Component Analysis (PCA) using JMP Genomics in SAS was performed, summarizing the major patterns of variation in a multi-locus data set. To improve the distribution of all five parameters for GWAS analyses, means were calculated using procedure GLIMMX in SAS, as previously described. The GWAS analyses were conducted using the software TASSEL 3.0 (Bradbury et al., 2007), with the Mixed Linear Model option, which considers both population structure and kinship. The extent of linkage disequilibrium (LD) was determined as the squared allele frequency correlation estimates (*r^2^*, Weir, 1996) and to measure the significance of *r^2^*. The expected *p*-value versus the observed *p*-value test statistics for the SSR markers were plotted (Q-Q plot) to assess the control of type I (false positive) errors under multiple run parameters, as previously published for RMC accessions (Bryant et al., 2011; Li et al., 2011, 2012). A marker-trait association was determined to be significant when the *p*-value was *p* < 0.01. Individual trials performed for each chilling tolerance trait were run as independent studies to account for environmental or year-to-year effects, and as means. Logarithm of odds (LOD) scores were calculated as –log10*p*.

### Identification of Potential Candidate Genes in the QTL Regions

Candidate genes at or near the QTLs reported in this study were identified in the QTARO database (Yonemaru et al., 2010). The candidate gene positions were updated to the most recent IRGSP 1.0 annotation using RiceXpro (Sato et al., 2011).

## Results and Discussion

### Assessment of Five Chilling Tolerance Indices in Different RMC Subpopulations

**FIGURE 2:**
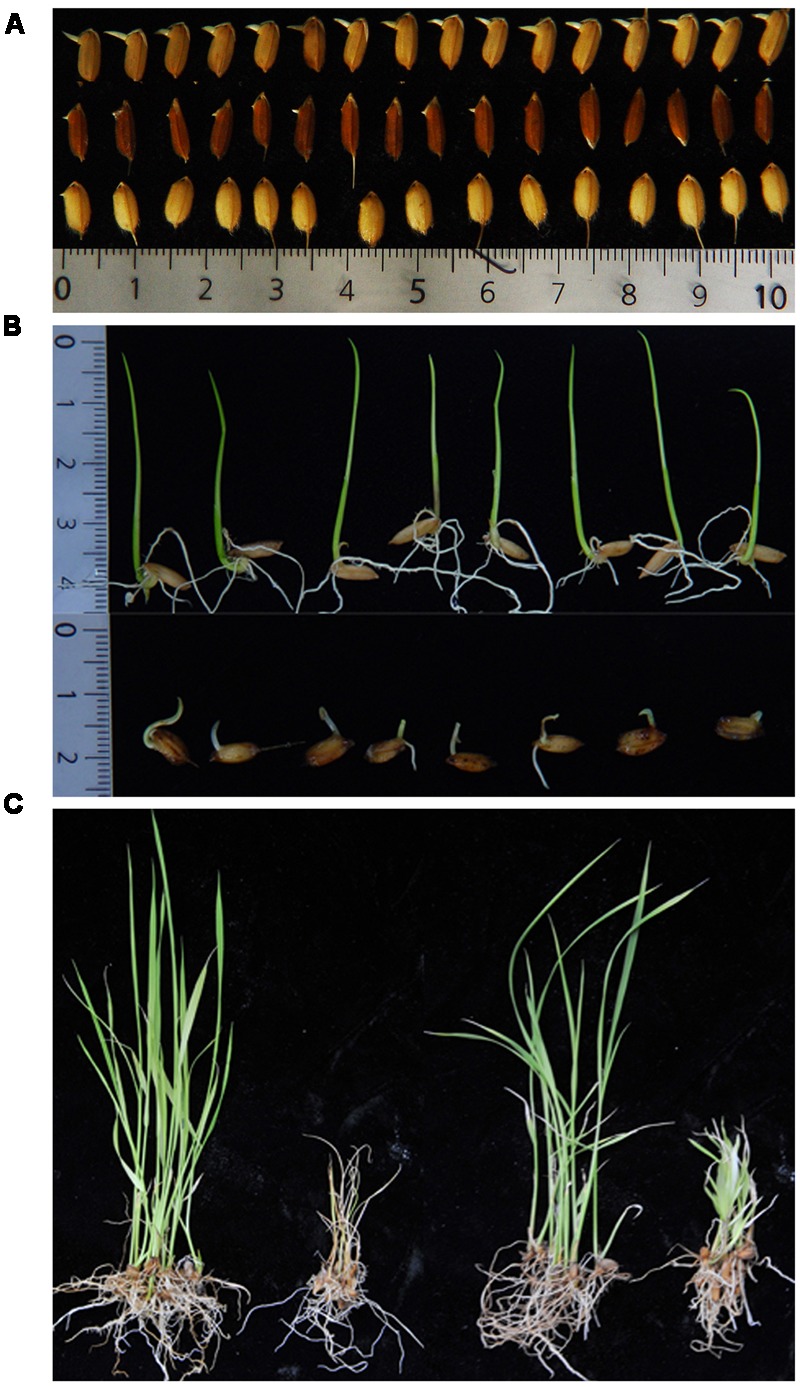
Examples of the five chilling tolerance indices assessed in this study. **(A)** Low Temperature Germinability (LTG) measures the percent germination of seeds after 30 days of incubation at 10 ± 1°C. First row, ∼100% LTG; second row, ∼50% LTG; third row, ∼10% LTG. **(B)** Plumule Growth after Cold Germination (PGCG) measures the growth rate of young seedlings at 28 ± 1°C after 30 days of germination at 10 ± 1°C. The first row illustrates high PGCG and the second row low PGCG. **(C)** Low Temperature Seedling Survivability (LTSS, left two plants), measures the survivability of 2-week-old seedlings after 1 week of incubation at 10 ± 1°C and 1 week of recovery growth at 28 ± 1°C. ∼100% LTSS (far left) vs. ∼0% LTSS (second from left). Plumule Growth after Cold (PGC, right two plants) measures the ability to resume growth at 28 ± 1°C after 1 week of incubation at 10 ± 1°C. ∼100% PGC (2-fold increase; second from right) vs ∼0% PGC (green but no growth; far right).

We assessed five phenotypic assays as potential quantitative trait loci (QTL) mapping indices reflecting cold stress scenarios rice seeds and seedlings might be exposed to during early developmental history (**Figure 2**). To address cold temperature scenarios for rice seeds, we developed the LTG and PGCG assays, and to address cold temperature scenarios for young seedlings, we developed the PGCG, LTSS, PGC, and overall LTS assays. For most indices, large differences between the *INDICA* and *JAPONICA* were observed. To associate the five chilling tolerance indices with the population structure of the RMC, we calculated for each subpopulation, a mean and standard error for the five indices, and compared the distributions to the generally more-chilling tolerant *temperate japonica* population using a Student’s *t*-test. Most subpopulations had similar LTG means, however, for *aromatic* and *aus* the values were slightly lower (**Table 1** and Supplementary Table S1). For the other four indices PGCG, LTSS, PGC, and LTS, *aus* and *indica* had the lowest values (*p* < 0.0001), in agreement with previous reports that *INDICA* accessions are less chilling tolerant than *JAPONICA* (Mackill and Lei, 1997; Lv et al., 2016). The means for cold tolerant *temperate japonica* and cold sensitive *indica* checks used as controls for each trial were in agreement with the mean of their respective subpopulation group (**Table 1**).

**Table 1:** Means and standard error for the five seedling chilling tolerance indices by the five rice subpopulation groups and the admixture of two or more subpopulations.

Subpopulation group	LTG^†,‡^	PGCG^†,‡^	LTSS^†,‡^	PGC^†,‡^	LTS^†,‡^
*Aus*	27.95 ± 4.27*	4.20 ± 0.32***	14.98 ± 1.77***	2.94 ± 1.30***	27.42 ± 2.80***
*Indica*	36.51 ± 2.88*	4.91 ± 0.22***	8.67 ± 1.46***	1.31 ± 0.71***	19.10 ± 2.14***
Admixture	45.10 ± 5.05	6.25 ± 0.35*	43.36 ± 6.66	21.70 ± 4.30**	36.74 ± 6.05***
*Aromatic*	5.01 ± 2.95***	5.94 ± 0.65	54.40 ± 7.80*	36.45 ± 17.43	70.35 ± 6.91
*Tropical japonica*	42.88 ± 3.63	5.89 ± 0.30**	67.09 ± 3.07*	27.81 ± 3.10*	68.04 ± 2.17*
*Temperate japonica*	45.24 ± 3.08	7.32 ± 0.22	75.92 ± 1.99	42.09 ± 3.48	77.07 ± 1.93
Cold resistant checks	70.25 ± 14.91	6.95 ± 0.65	90.15 ± 3.38	49.89 ± 13.57	69.52 ± 9.91
Cold sensitive checks	71.52 ± 12.37	0.46 ± 0.00	0.00 ± 0.00	0.00 ± 0.00	6.81 ± 5.27

*Means of the subpopulation groups are compared to the *temperate japonica* subpopulation, using a Student’s *t*-test. ^†^Five seedling chilling tolerance indices: low temperature germinability (LTG), plumule growth rate after cold germination (PGCG), low temperature seedling survivability (LTSS), plumule recovery growth after cold exposure (PCG), and low temperature survival (LTS). See Supplementary Table S1 for scores of individual mini-core accessions. ^‡^Student’s *t*-test relative to *temperate japonica* calculated in Microsoft Excel using the *T*-TEST function (2-tailed, unequal variance). ^∗^*P* < 0.05; ^∗∗^*P* < 0.001; ^∗∗∗^*P* < 0.0001.*

To determine how the means of different chilling tolerance indices for each RMC accession were compared to each other, pairwise Pearson’s correlation analysis was conducted (**Table 2**). This showed LTG and PGCG were not (LTG) or weakly (PGCG) correlated with other indices while the LTSS, PGC, and LTS indices were highly correlated with each other. This suggests that the LTG and PGCG indices have a relatively unique genetic program while LTS, LTSS, and PGC share genetic pathways. PGCG most likely correlated with both LTG and the seedling tolerance indices due to its germination and coleoptile/plumule growth components. This implies rice accessions that germinate well in cold temperatures do not necessarily survive well at the young seedling stage and *vice versa* thus suggesting the genetic pathways controlling cold temperature germinability and survival at the germination stage are fundamentally different from those controlling chilling tolerance at the young seedling stages. This is in agreement with a previous report (Ranawake et al., 2014).

**Table 2:** Pearson’s correlation coefficients between the five different rice seedling chilling tolerance indices evaluated in this study.

Trait^†^	LTG	PGCG	LTSS	PGC
PGCG	0.369^∗∗∗^			
LTSS	0.162	0.384^∗∗∗^		
PGC	0.146	0.313^∗∗∗^	0.805^∗∗∗^	
LTS	0.105	0.331^∗∗∗^	0.894^∗∗∗^	0.676^∗∗∗^

*^†^Five seedling chilling tolerance indices: low temperature germinability (LTG), plumule growth rate after cold germination (PGCG), low temperature seedling survivability (LTSS), plumule recovery growth after cold exposure (PCG), and low temperature survival (LTS). **^∗∗∗^***P* < 0.0001.*

Taken together, an assessment of the five chilling tolerance indices indicates the different subpopulations of the RMC collection have significantly different chilling tolerance abilities, potentially reflecting different genetic mechanisms. This suggests QTL mapping has the potential to uncover subpopulation specific loci with either positive or negative effects on the described chilling tolerance indices.

### GWAS Mapping of Five Chilling Tolerance Indices

**FIGURE 3:**
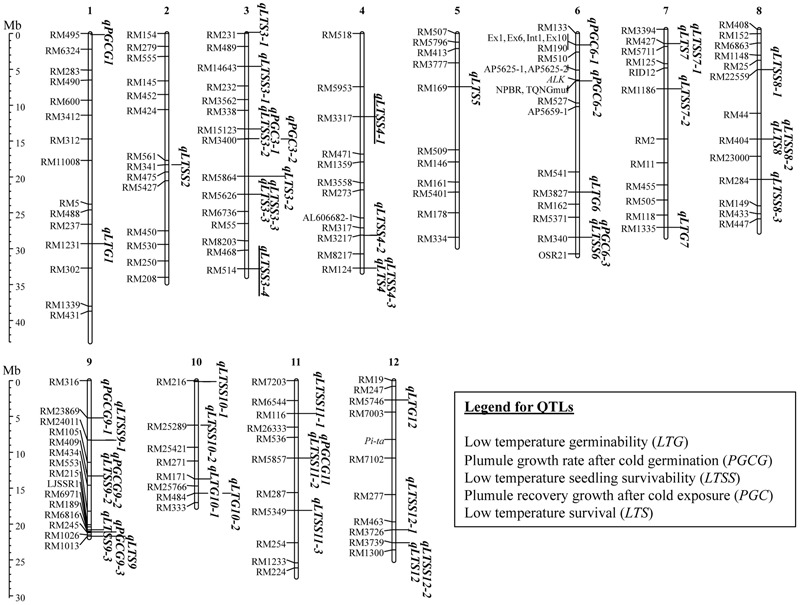
Quantitative trait loci (QTL) locations based on genome wide association study (GWAS) mapping analysis using the rice mini-core collection (RMC) as reported in **Table 3**. The QTL, *qLTSS3-4* and *qLTSS4-1* (underlined) are novel, whereas the other QTL overlap with or are within 1.5 Mb of previously published chilling tolerance QTL (Supplementary Table S2). Details of the individual QTL are in **Table 3**. Markers are further described in Bryant et al. (2011), and the figure was created in MapChart (Voorrips, 2002).

To identify chilling tolerance QTL associated with the five assays, a genome wide association study (GWAS) mapping was conducted after removal of rare allele markers at 3% or less. Based on previous GWAS mapping assessments using the relatively skewed population structure of the RMC (Bryant et al., 2011, 2013; Li et al., 2011, 2012; Jia et al., 2012) or other small rice collections (Pan et al., 2015), a cutoff *p*-value of 0.01 was chosen, which identified 39 markers that associated with different chilling-tolerance QTL across all 12 chromosomes, eight of which overlapped with two indices (**Figure 3**). We obtained six *LTG*-QTL, five *PGCG*-QTL, 24 *LTSS*-QTL, five *PGC*-QTL, and eight *LTS-*QTL (**Table 3**). Of the 39 associated chromosomal regions, 37 were within 1.5 megabase pairs (Mb) of previously published rice chilling tolerance QTL, while two novel QTL, *qLTSS3-4* and *qLTSS4-1*, were identified (Supplementary Table S2). Thus, GWAS mapping using the RMC collection and five chilling tolerance indices identified 37/39 = 95% of previously published chilling tolerance QTL that were identified with bi-parental mapping populations (18 QTL; reviewed in da Cruz et al., 2013; Zhang et al., 2014a) or recent GWAS mapping using different rice diversity panels (32 QTL; Fujino et al., 2015; Pan et al., 2015; Lv et al., 2016; Wang et al., 2016a; Shakiba et al., 2017).

**Table 3:** Summary of the QTL identified by GWAS mapping for five different rice seedling chilling tolerance indices.

QTL	Trial no.^†^	*p-*value (x 10^-^^3^)	LOD value^‡^	Chromosome	Position (Mb)	Associated marker	Marker *R2* (%)^#^
***Low temperature germinability (LTG)***							
*qLTG1*	1	0.74	3.13	1	29.5	RM1231	11.90
	3	2.73	2.57	1	29.5	RM1231	10.44
	combined	1.13	2.95	1	29.5	RM1231	11.45
*qLTG6*	3	1.63	2.79	6	22.3	RM3827	12.97
	combined	1.35	2.87	6	22.3	RM3827	13.20
*qLTG7*	2	2.81	2.55	7	28.3	RM1335	11.25
*qLTG10-1*	2	3.77	2.42	10	19.1	RM171	6.30
	combined	10.53ˆ!	1.98	10	19.1	RM171	5.15
*qLTG10-2*	3	4.00	2.40	10	21.1	RM484	5.99
	combined	6.44	2.19	10	21.1	RM484	5.48
*qLTG12*	2	0.26	3.58	12	5.1	RM5746	9.47
***Plumule growth rate after cold germination (PGCG)***							
*qPGCG1*	1	4.53	2.34	1	0.2	RM495	5.38
*qPGCG9-1*	1	3.55	2.45	9	6.3	RM23869	7.91
*qPGCG9-2*	1	6.71	2.17	9	14.4	RM409	5.98
*qPGCG9-3*	1	7.04	2.15	9	22.2	RM6816	3.58
*qPGCG11*	1	6.98	2.16	11	11.9	RM5857	13.34
***Low temperature seedling survivability (LTSS)***							
*qLTSS2*	4	6.56	2.22	2	19.4	RM341	5.81
*qLTSS3-1*	1	0.15	3.82	3	7.1	RM14643	9.28
*qLTSS3-2*	1	4.68	2.33	3	15.8	RM15123	12.56
	2	8.61	2.07	3	15.8	RM15123	10.89
*qLTSS3-3*	2	7.51	2.12	3	24.9	RM5626	5.55
	combined	0.29	3.54	3	24.9	RM5626	5.56
*qLTSS3-4*	combined	10.43ˆ!	1.98	3	35.3	RM514	6.21
*qLTSS4-1*	1	0.15	3.84	4	13.6	RM3317	12.32
	2	0.30	3.52	4	13.6	RM3317	10.75
	combined	0.93	3.03	4	13.6	RM3317	8.51
*qLTSS4-2*	2	7.55	2.12	4	30.1	RM3217	6.45
*qLTSS4-3*	combined	2.72	2.57	4	34.7	RM124	3.95
*qLTSS6*	1	6.45	2.19	6	28.6	RM340	8.51
*qLTSS7-1*	3	7.33	2.14	7	2.7	RM427	1.98
*qLTSS7-2*	4	5.61	2.25	7	9.0	RM1186	6.31
*qLTSS8-1*	combined	4.87	2.31	8	5.7	RM22559	4.87
*qLTSS8-2*	2	6.64	2.18	8	15.4	RM404	11.04
	5	0.64	2.20	8	15.4	RM404	5.07
*qLTSS8-3*	4	4.50	2.35	8	21.0	RM284	6.04
*qLTSS9-1*	5	0.54	2.27	9	9.4	RM24011	8.14
*qLTSS9-2*	combined	10.85ˆ!	1.97	9	14.4	RM409	4.25
*qLTSS9-3*	4	7.77	2.11	9	21.9	RM6971	10.77
*qLTSS10-1*	2	9.16	2.04	10	5.4	RM216	6.27
	combined	5.68	2.25	10	5.4	RM216	4.65
*qLTSS10-2*	1	0.64	3.19	10	11.6	RM25289	9.98
*qLTSS11-1*	4	3.15	2.50	11	5.7	RM116	5.09
*qLTSS11-2*	2	3.35	2.48	11	11.9	RM5857	13.00
*qLTSS11-3*	1	8.51	2.07	11	19.2	RM5349	8.35
*qLTSS12-1*	4	10.81ˆ!	1.97	12	23.2	RM3726	9.04
	combined	0.70	3.15	12	23.2	RM3726	10.80
*qLTSS12-2*	3	0.37	3.43	12	25.0	RM3739	3.91
	combined	9.55	2.02	12	25.0	RM3739	5.40
***Plumule recovery growth after cold exposure (PGC)***							
*qPGC3-1*	combined	10.51ˆ!	1.98	3	15.8	RM15123	10.65
*qPGC3-2*	combined	2.51	2.60	3	17.2	RM3400	11.41
*qPGC6-1*	combined	9.91	2.00	6	1.8	Ex_6	2.96
*qPGC6-2*	combined	4.81	2.32	6	6.8	NPBR	3.57
*qPGC6-3*	combined	1.48	2.83	6	28.6	RM340	10.02
***Low temperature survival (LTS)***							
*qLTS3-1*	2	9.13	2.04	3	2.5	RM231	8.79
*qLTS3-2*	1	5.85	2.23	3	24.9	RM5626	6.40
	combined	2.39	2.62	3	24.9	RM5626	7.09
*qLTS4*	1	2.14	2.67	4	34.7	RM124	5.86
	combined	0.01	5.00	4	34.7	RM124	10.00
*qLTS5*	1	5.04	2.30	5	7.5	RM169	10.64
*qLTS7*	combined	10.71ˆ!	1.97	7	2.7	RM427	4.21
*qLTS8*	combined	0.46	3.34	8	15.4	RM404	16.55
*qLTS9*	1	6.52	2.19	9	22.8	RM1013	4.86
*qLTS12*	combined	3.58	2.45	12	25.0	RM3739	7.30

*All QTL below the *p*-value = 0.01 are included. ^†^QTL identified from the individual trials or combined across all trials for the particular index. ^‡^The LOD value is the logarithm of odds (-log10*p*). ^#^Marker effects as identified by TASSEL 3.0 (Bradbury et al., 2007). ^!^Value very close to *p* = 0.01 cutoff.*

Of the 48 QTL at 39 locations, 30 QTL occupied only one location while 18 QTL shared nine sites: one site on chr. (chromosome) 3 was shared by *qLTSS3-2* and *qPGC3-1*, another site on chr. 3 by *qLTS3-2* and *qLTSS3-3*, one site on chr. 4 by *qLTS4* and *qLTSS4-3*, one site on chr. 6 by *qLTSS6* and *qPGC6-3*, one site on chr. 7 by *qLTS7* and *qLTSS7-1*, one site on chr. 8 by *qLTS8* and *qLTSS8-2*, one site on chr. 9 by *qLTSS9-2* and *qPGCG9-2*, one site on chr. 11 by *qLTSS11-2* and *qPCGC11*, and one site on chr. 12 by *qLTS12* and *qLTSS12-2* (**Figure 3** and **Table 3**). Interestingly, all six *LTG-*QTL were at sites not shared by QTL identified by the other indices while five of the eight *LTS*-QTL (62.5%) were shared. For the *PGC*-QTL and *PGCG*-QTL, two of their respective five QTL (40%) were shared, and for *LTSS*-QTL, 9 of the 24 (38%) were shared. In agreement with the indices correlation analysis (**Table 2**), this further suggests *LTG-*QTL associate with relatively unique genetic mechanisms while *LTSS-, PGC-, LTS-*, and to some extent *PGCG*-QTL, share overlapping or converging genetic mechanisms. Future fine mapping studies will determine whether overlapping QTL associate with one or two genetic factors. Although the RMC was previously used for GWAS based QTL mapping of agricultural traits (Bryant et al., 2011, 2013; Li et al., 2011, 2012; Jia et al., 2012; Wang et al., 2016b), to the best of our knowledge, this work is the first GWAS based mapping analysis to identify chilling tolerance QTL using the 202 *O. sativa* accessions of the RMC. What follows is an individual discussion of each chilling tolerance index and its associated QTL.

### Low Temperature Germinability (LTG) Index and QTL

The growth chamber based LTG assay was designed to mimic the planting of rice either by drill-seeding or water-seeding, as previously described. Earlier studies demonstrated the genetic basis for LTG (Fujino et al., 2008, 2015; Shakiba et al., 2017), thus we expected RMC accessions with superior chilling tolerance to germinate better in cold water or in soil under cool spring temperatures than those with inferior tolerance. In agreement with this, different RMC accessions had reproducibly distinct LTG indices, as shown in **Figure 2A**, which illustrates the difference in germination observed in this study.

The mean Pearson’s correlation coefficient between three trials was *r* = 0.539 (0.363–0.950; *p* < 0.001). Of the six QTL uncovered in this study (**Figure 3**), three were near previously identified *LTG*-QTL on chr. 7 reported as *qCTGERM7-5* (Shakiba et al., 2017), on chr. 10 reported as *qLTG10* (Fujino et al., 2015), and on chr. 12 reported as *qLTG12b* (Fujino et al., 2015), while the other three were near other types of previously mapped chilling tolerance QTL (Supplementary Table S2). All six *LTG*-QTL had genes previously annotated for cold and other abiotic stress tolerance associated with them within 1.5 Mb of the marker associated with the particular QTL (Supplementary Table S3). An interesting candidate gene for *qLTG10-2* may be *MYBS3*, previously shown to confer chilling tolerance when overexpressed in rice (Su et al., 2010). Taken together, the LTG assay and GWAS based mapping analysis uncovered six robust *LTG*-QTL, which can be used for marker assisted breeding and gene discovery. Because LTG values were relatively similar between different subpopulations, both *INDICA* and *JAPONICA* subspecies are expected to have alleles contributing to superior LTG abilities, in agreement with the recent identification of *INDICA* specific *LTG* QTL (Shakiba et al., 2017).

### Plumule Growth Rate after Cold Germination (PGCG) Index and QTL

The PGCG assay was developed to determine whether germination at low temperatures had a quantitative effect on subsequent growth and development of seedlings at warmer temperatures, because we noticed that some accessions with good LTG indices did not grow well after a temperature shift to 28°C and *vice versa*. For the direct seeding method, this situation might address a realistic scenario in which rice seeds may experience an extended period of cold during germination followed by warm, growth promoting temperatures. Interestingly, while many accessions developed a robust plumule, others showed only minor elongation of the coleoptile and stopped growth altogether during the 4-day assay period (**Figure 2B**). Because there was only a modest correlation (*r* = 0.369, *p* < 0.001) between PGCG and LTG (**Table 2**), *PGCG*-QTL were expected to specifically associate with seedling development after cold temperature germination. In agreement with this, although the PGCG index had a 1-month germination period at 10°C, similar to LTG, there was no overlap between *LTG*- and *PGCG*-QTL (**Figure 3**), suggesting the two indices are controlled by different genetic pathways. Of the five *PGCG* QTL revealed in this study, four were in the approximate location of previously mapped seedling chilling tolerance QTL (Supplementary Table S2) on chr. 1 identified as *qIW-1* (Ji et al., 2010) and *qCST1-1* (Liu et al., 2013), and on chr. 9 identified as *qCTS9-7* (Wang et al., 2016a), *qCTS-9* (Zhang et al., 2014b), and “*Survival Rate after Natural Chilling Stress*” Locus 100 (Lv et al., 2016), which, at least in part, might be due to the coleoptile/plumule recovery growth part of the PGCG index. It is noteworthy that *qPGCG1* is only 3,000 base pairs (bp) away from *OsCOIN* (Supplementary Table S3), previously reported to confer chilling tolerance in rice (Liu et al., 2007). Of particular interest is also *qPCGC9-2*, which is near *OsWRKY76* previously shown to increase cold tolerance when overexpressed (Yokotani et al., 2013), and 160,000 bp away from Os09g24440 (Supplementary Table S3), a putative subunit of TFIID recently shown to be the functional gene underlying *qCTS-9* and to enhance chilling cold tolerance when overexpressed (Zhao et al., 2017). Allelic differences at some of those genes may thus contribute to different PGCG indices.

Taken together, PGCG is a useful index to select accessions with a superior ability to recover from cold temperature germination by adjusting their genetic pathway and metabolism to a growth promoting temperature. However, another common practice for the water-seeding method is to pre-germinate seeds at a high temperature for 1 day before planting (Zhang et al., 2014a), resulting in slightly emerged coleoptiles. Such seeds might experience cold stress after planting, which should be tested in future experiments to determine whether this scenario overlaps with the PGCG or other traits.

### Low Temperature Seedling Survivability (LTSS) Index and QTL

The LTSS assay was developed to mimic the traditional method of transplanting young seedlings, because the young seedlings might be temporarily exposed to suboptimal or even lethal water or ambient temperatures during the transplanting process in the early spring. Seedlings of RMC accessions with superior chilling tolerance ability were expected to have high survivability index after a transient cold exposure while seedlings of accessions with inferior abilities were expected to have low survivability index. In agreement with this, LTSS for different accessions ranged from 0% to almost 100%, indicating quantitative genetic differences between cultivars (**Figure 2C**). Although we observed seedlings of numerous RMC cultivars exhibited a rolled leaf phenotype during the cold treatment, suggesting water stress, with few exceptions most of them remained green and did not wilt during the 1-week incubation at 10°C. In contrast, severe wilting and death occurred in chilling sensitive seedlings during the first few days of recovery growth, while accessions with superior chilling tolerance abilities remained green and most of them resumed growth. Thus, the LTSS assay is a robust assessment of seedling chilling tolerance and may be a good predictor of the field performance for the individual rice accessions when young seedlings are transplanted in the early spring.

**FIGURE 4:**
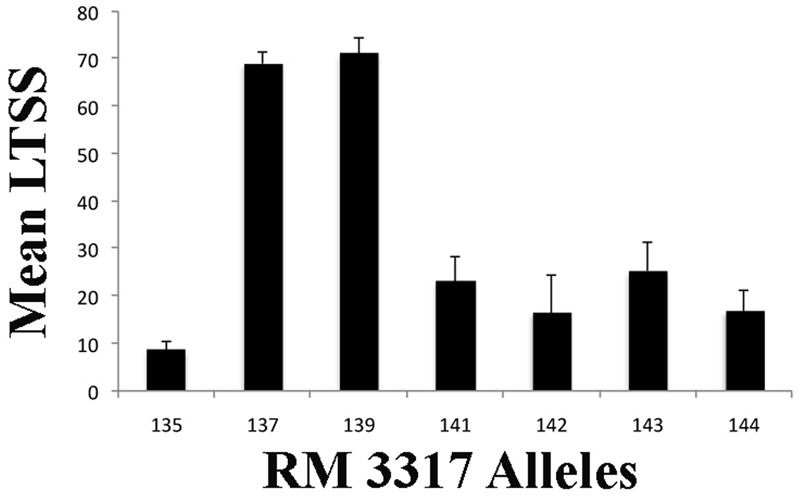
Association of genotypic effects of RM3317 alleles with mean LTSS scores at the *qLTSS4-1* locus. Shown is the mean low temperature seedling survival (LTSS) for the RMC accessions having the same RM3317 allele. Alleles 137 and 139 are predominantly found in *JAPONICA* accessions and alleles 135, 141, 142, 143, and 144 are predominantly found in *INDICA* accessions (Supplementary Table S4). Error bars indicate the standard error of LTSS for the accessions having a particular allele.

The five LTSS trials yielded relatively similar results with a mean Pearson’s coefficient of *r* = 0.679 (0.543–0.806; *p* < 0.0001), thus the trials were also combined for GWAS based QTL mapping (**Table 3**). Of the 24 QTL uncovered in this study (**Figure 3**), 22 were near previously identified QTL while two, *qLTSS4-1* and *qLTSS3-4*, were novel (Supplementary Table S2). Interestingly, *qLTSS4-1* was obtained three times with high LOD scores (**Table 3**) and might be related to the particular collection of accessions composing the RMC. Moreover, while most *LTSS*-QTL had previously annotated cold and other stress tolerance genes associated with them within 1.5 Mb of the marker (Supplementary Table S3), *qLTSS4-1* did not, suggesting that it is a truly novel, seedling chilling tolerance QTL. Because *qLTSS4-1* marker alleles with positive genotypic effects (alleles 137 and 139; **Figure 4** and Supplementary Table S4) were almost exclusively associated with the *JAPONICA* and alleles with negative effects almost exclusively with *INDICA* (alleles 135, 141, 142, 143, and 144; **Figure 4** and Supplementary Table S4), *qLTSS4-1* may be an interesting new QTL useful for marker assisted breeding and novel gene discovery. In addition to validating our GWAS mapping results, many of the identified RMC *LTSS*-QTL that occur near previously mapped seedling chilling tolerance QTL help narrow down the QTL region and provide further support of locations where chilling tolerance genes occur. Of particular interest are the previously mentioned *OsWRKY76* and Os09g24440 near *qLTSS9-2*, and *qLTSS4-3*, as it is 280,000 bp away from *OsCAF1B*, a gene involved in RNA processing previously reported to be cold induced (Chou et al., 2014; Supplementary Table S3).

### Plumule Recovery Growth after Cold Exposure (PGC) Index and QTL

The PGC assay was developed, because we noticed that although some RMC accessions had relatively good LTSS values, as defined by retaining apparently healthy looking leaves after the cold period, they did not resume growth during the recovery period at higher temperatures (**Figure 2C**). Seedlings remained green, indicating that the existing leaves were chilling tolerant, but cell division and elongation activity at the base of leaves or in the shoot apical meristem appeared to be compromised after the cold treatment. After the cold treatment and recovery periods, mean plumule lengths ranged from 0% for accessions that died or stopped developing to approximately 100% (2-fold increase) for accessions that doubled the length of their plumule during recovery, indicating quantitative genetic differences between the accessions. Thus, PGC was a suitable index for mapping QTL associated with this leaf growth recovery parameter.

The mean Pearson’s correlation between three PGC trials was *r* = 0.495 (0.435–0.571; *p* < 0.001), and all replicates were combined for GWAS mapping. All five *PGC*-QTL uncovered in this study were near previously mapped seedling chilling tolerance QTL (Supplementary Table S2), two on chr. 3 reported as “*Ratio of Electrolyte Leakage under 3 Day Cold Shock Stress to Normal Condition*” Loci 36 and 37 (Lv et al., 2016), and three on chr. 6 reported as *COLD4* (Ma et al., 2015) or *qCST6(1)* (Ranawake et al., 2014), *qCTS6-2* (Wang et al., 2016a), and *qLTSSvR6-1* (Pan et al., 2015) or *qCST6* (Liu et al., 2013). Also, several previously annotated cold stress tolerance genes were within 1.5 Mb of these *PGC*-QTL (Supplementary Table S3). Of particular interest might be *OsDREB1C* associated with *qPGC6-1*, which was previously shown to play a critical role in controlling cold stress regulons (Chawade et al., 2013). Although there was a good correlation between mean LTSS and PGC indices (*r* = 0.805; *p* < 0.0001; **Table 2**), the PGC index may additionally be controlled by a distinct genetic pathway, because only two of the five *PGC*-QTL overlapped with *LTSS*-QTL (**Figure 3**). Therefore, although the stress regime for seedlings was the same, LTSS and PGC appear to assess, at least in part, two different challenges young seedlings may face during cold stress, which are, respectively: maintenance of cellular homeostasis and metabolism during the cold and recovery periods (photosynthesis and respiration for LTSS), and resumption of vegetative growth and development activities during the recovery period (mitotic activities in the meristem or at the base of leaves for PGC).

### Low Temperature Survival (LTS) Index and QTL

The LTS assay was developed to mimic the developmental history of a rice seed germinating under low temperature stress and developing into a young seedling after ambient temperatures rise, then temporarily experiencing additional cold stress at the young seedling stage. This was done to assess whether the low temperature survivability of seedlings was either positively or negatively affected by a preceding period of low temperature germination, because we hypothesized that some rice accessions can “acclimate” to chilling stress during the low temperature germination period, resulting in higher LTS than LTSS indices. The LTS assay specifically addressed chilling acclimation, because only seedlings that survived the 2-week recovery period following low temperature germination were used for the low temperature seedling survival measurements. Remarkably, for both *aus* and *indica* subpopulations, the mean LTS was 2-fold higher than mean LTSS (**Table 1**). This suggested the *INDICA* RMC accessions had a certain degree of chilling acclimation potential during cold temperature germination, in agreement with our hypothesis based on the paired Student’s *t*-tests indicating both subpopulations had significantly higher LTS than LTSS indices (*indica p* = 3.12 × 10^-9^; *aus p* = 7.31 × 10^-6^), while the other subpopulations did not (*p* > 0.3). However, even though a similar number of approximately 25 healthy seedlings were used for each LTSS and LTS trial (see Materials and Methods), we cannot rule out the possibility that the elimination of seeds with poor PGCG indices artificially increased LTS values compared to LTSS, but we consider this less likely, because it is not clear why this would only affect the *INDICA* and not the *JAPONICA* subspecies, nor the admixture subpopulation (**Table 1**).

The Pearson’s correlation between the two trials was *r* = 0.614 (*p* < 0.0001), thus the trials were combined for GWAS mapping. Of the eight *LTS-*QTL uncovered in this study, five were near previously mapped low temperature germination QTL (Supplementary Table S2), one on chr. 3 reported as *qLTG3c* (Fujino et al., 2015), one on chr. 4 reported as *qLTG4* (Fujino et al., 2015), one on chr. 5 reported as *qLVG5* (Han et al., 2006), one on chr. 7 reported as *qLVG7-1* and *qCIVG7-2* (Han et al., 2006), and one on chr. 12 reported as *qCTGERM12-2* (Shakiba et al., 2017), suggesting that genes associated with them might be involved in controlling low temperature acclimation during the germination period. In addition, three of the QTL were near previously mapped seedling chilling tolerance QTL (Supplementary Table S2), one on chr. 3 reported as *qCTS3-5* (Wang et al., 2016a), one on chr. 8 reported as “*Fresh versus Dry Biomass Ratio after Natural Chilling Stress*” Locus 84 (Lv et al., 2016), and one on chr. 9 reported as “*Survival Rate after Natural Chilling Stress*” Locus 100 (Lv et al., 2016). Eight of the nine QTL had previously annotated cold (*OsCAF1B*; Chou et al., 2014) and other abiotic stress tolerance genes such as the ABA receptor *OsPYL/RCAR5* (Kim et al., 2014) within 1.5 Mb of the QTL markers (Supplementary Table S3), which might associate, at least in part, with genes that have a positive effect on chilling acclimation. The type of chilling acclimation potential addressed here is associated with low temperature germination, which might be different from exposing young seedlings first to a mild chilling temperature followed by a harsh cold temperature treatment previously shown to have a positive effect for some accessions of both the *INDICA* and *JAPONICA* (Mao and Chen, 2012).

## Conclusion

In this study, five chilling tolerance indices (**Figure 2**) reflecting the early life history of rice seeds and young seedlings during springtime planting in temperate climates were developed and assessed using the USDA RMC collection. Significant differences between RMC accessions were observed for all five chilling tolerance indices measured, with a general tendency for the accessions from *JAPONICA* to have better chilling tolerance abilities than those from *INDICA* (**Table 1** and Supplementary Table S1). However, *INDICA* accessions had chilling acclimation potential during germination while those from *JAPONCIA* did not. This indicated that both the RMC accessions and the five seedling chilling tolerance indices were suitable for GWAS based mapping of chilling tolerance QTL, yielding 48 QTL at 39 chromosome regions (**Figure 3** and **Table 3**). There was no overlap between germination and seedling stage QTL (**Figure 3**), nor a good correlation between the germination and seedling stage indices (**Table 2**), suggesting the genes controlling those two general indices belong to fundamentally different genetic pathways. Moreover, this study identified two novel *LTSS*-QTL, *qLTSS3-4* and *qLTSS4-1*, which appear to be RMC specific because they were not revealed in previous QTL studies. The novel *qLTSS4-*1 QTL has *JAPONICA* specific alleles with positive genotypic effects (**Figure 4** and Supplementary Table S4), which might be useful for marker assisted breeding programs and the discovery of potentially novel chilling tolerance genes.

## Author Contributions

MS conceived the study; MS and GE wrote the paper; MS, AW, and NS performed most of the phenotyping trials; AJ performed the GWAS mapping analyses and helped MS analyze the results; CC suggested experiments and provided resources; GE and YS helped interpret the data, DB performed statistical analyses. All authors contributed to improving the manuscript.

## Conflict of Interest Statement

The authors declare that the research was conducted in the absence of any commercial or financial relationships that could be construed as a potential conflict of interest.

## Footnotes

1 www.usclimatedata.com
